# Supplementary material for: Partially native intermediates mediate misfolding of SOD1 in single-molecule folding trajectories
Source: Nat Commun. 2017 Dec 1;8:1881. doi: 10.1038/s41467-017-01996-1 (PMC5709426; doi:10.1038/s41467-017-01996-1)
Supplement: Supplementary file 1 — Supplementary Information [file 41467_2017_1996_MOESM1_ESM.pdf]

SUPPLEMENTARY INFORMATION

SUPPLEMENTARY FIGURES

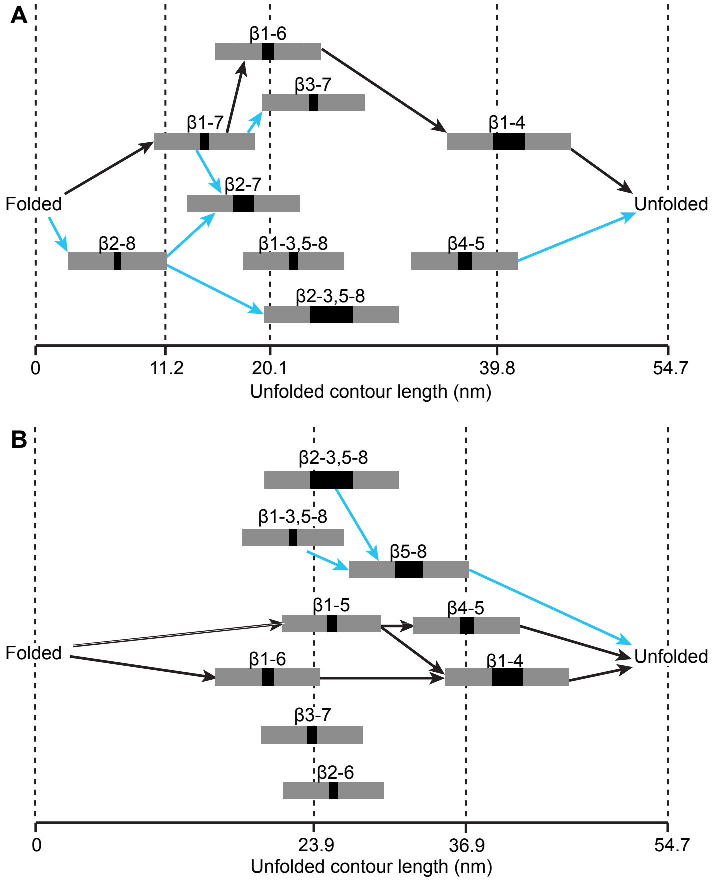

Supplementary Figure 1: Representative network analyses identifying possible intermediate structures from FECs. For each intermediate, all possible substructures consistent with the observed cumulative ΔL_c are shown (labeled with the strands that remain folded), displaying the range of lengths predicted from structural models (black) and allowing for up to 4 nm of mismatch between predicted and observed ΔL_c owing to experimental error (grey). Note that some of the structures have two disjunct folded regions (e.g. β2-3,5-8 and β1-3,5-8). Arrows indicate viable structural transitions, with non-productive paths unable to connect the folded and unfolded states shown in cyan. (A) For this FEC, only one possible pathway is consistent with the sequence of lengths observed: F → β1-7 → β1-6 → β1-4 → U. (B) Here, three pathways are consistent with the observed lengths, two of them (F → β1-6 or β1-5 → β1-4 → U) being part of the minimal model in Fig. 3B and the third (F → β1-5 → β4-5 → U) being part of a rare pathway not included in the model.

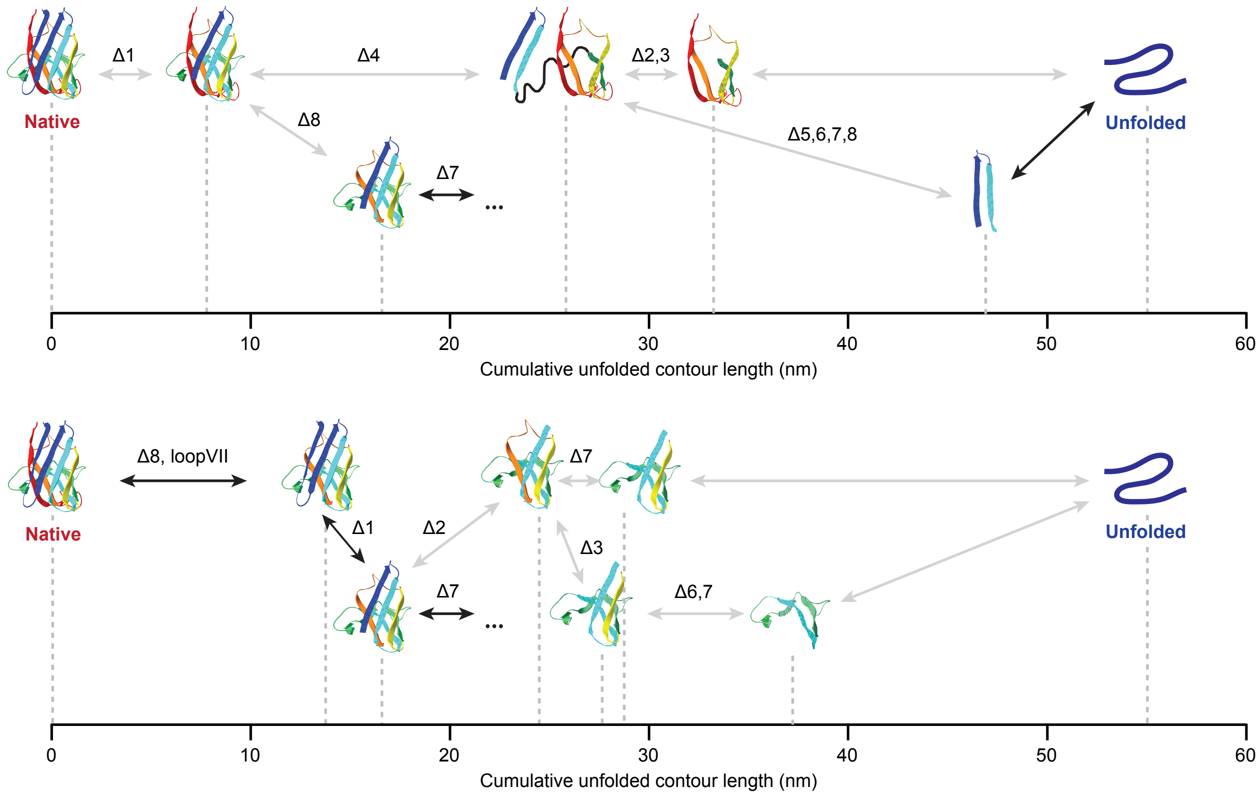

Supplementary Figure 2: Examples of minority pathways not included in minimal model. Additional pathways were identified in the network analysis but not included in the minimal model because they were rarely needed to explain the observed length changes in FECs (only a few percent of the time). The transitions between intermediates in some of these minority pathways are illustrated (grey arrows). Transitions that also occur in the minimal model are shown as black arrows. Ellipses represent places where the pathways return to the minimal model.

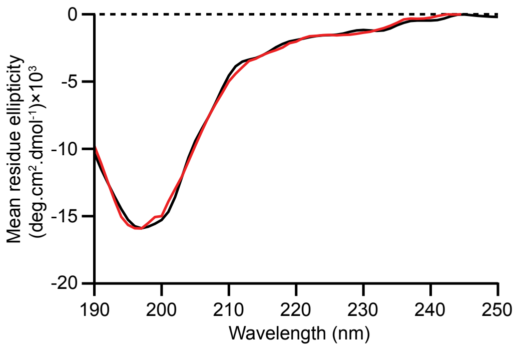

Supplementary Figure 3: CD spectrum of β2-3 peptide. The CD spectrum of the peptide (black) is consistent with spectra previously reported for β-hairpins.[13,14] Analysis of the secondary-structure content of the peptide by fitting with BeStSel[15] (red) finds roughly 37% β-sheet, 15% turn, and 47% irregular/loops, consistent with the structure expected from the hairpin formed by strands 2 and 3 of SOD1 (44% β-sheet, 20% turn, 36% unstructured).

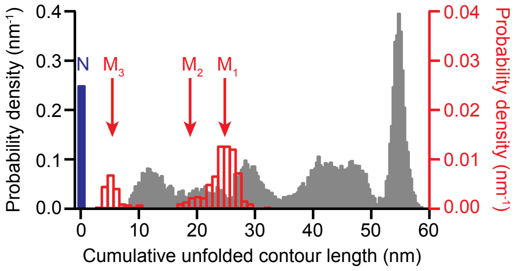

Supplementary Figure 4: Unfolded contour length of misfolded states compared to on-pathway intermediates. The most common misfolded states, M_1 and M_3, occur at contour lengths that are distinct from intermediates observed during the unfolding of native SOD1 (grey); M_2 is degenerate with the on-pathway intermediate I_6. All misfolded states have lifetimes much longer than any of the on-pathway intermediates.

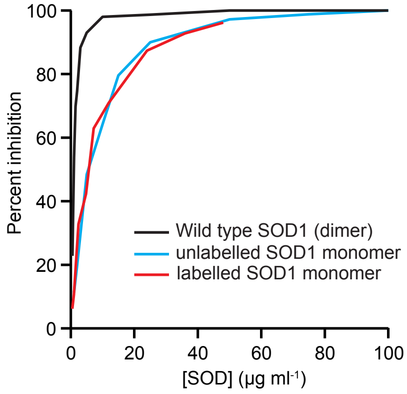

Supplementary Figure 5: Activity of SOD1 constructs. The activity of the SOD1 monomer was measured before (cyan) and after (red) functionalization of constructs with DTDP, showing no change. Monomer activity was lower than dimer activity (black), as expected.

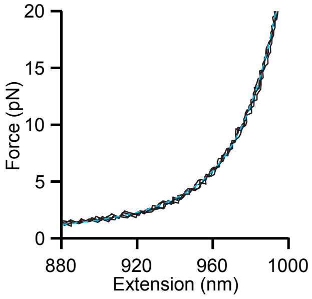

Supplementary Figure 6: FECs of DNA handles only. FECs of DNA handles only (black) show no rips and fit well to a simple worm-like chain model (cyan dashed line).

SUPPLEMENTARY TABLES

Supplementary Table 1: Possible substructures and their expected cumulative ΔL_c. All the substructures used to determine potential structures for the intermediates in the FECs are listed along with their expected cumulative ΔL_c, measured with respect to the native state. The range of values represents the variation between structural models.

Substructure	Range of expected cumulative ΔL_c (nm)	Substructure	Range of expected cumulative ΔL_c (nm)
**β2-8**	[6.7, 7.3]	**β4-7**	[26.2, 28.6]
**β1-7**	[14.3, 15.0]	**β3-6**	[27.9, 28.4]
**β2-7**	[17.0, 18.8]	**β2-5**	[28.5, 29.5]
**β1-6**	[19.4, 20.5]	**β5-8**	[31.2, 33.6]
**β1-3,β5-8**	[21.9, 24.1]	**β4-5**	[36.7, 37.9]
**β3-7**	[23.5, 24.3]	**β1-4**	[39.6, 42.3]
**β1-5**	[25.2, 26.0]	**β1-3**	[44.3, 44.9]
**β2-6**	[25.4, 26.1]	**β2-4**	[43.9, 46.0]
**β1-2,β5-8**	[25.0, 27.7]	**β1-2**	[46.8, 48.5]
**β2-3,β5-8**	[23.7, 27.4]	**β2-3**	[46.6, 48.1]

Supplementary Table 2: Sequence of monomeric SOD1 expression construct.

```
ATGAGCAGCCATCACCATCACCATCACGAAAACCTGTATTTTCAGAGCCTGTGTACCCCGAGCCGT
GGCGGTGGCGGTAGCGCGACCAAAGCCGTGGCGGTGCTGAAAGGCGACGGCCCAGTGCAGGGCATC
ATCAACTTCGAGCAGAAAGAAAGCAACGGCCCGGTGAAGGTGTGGGGCAGCATTAAAGGTCTGACC
GAAGGCCTGCATGGTTTCCATGTTCATGAGGAGGAGGATAATACAGCAGGCTGTACCAGCGCAGGT
CCGCACTTTAACCCGCTGTCCCGTAAACACGGTGGGCCAAAGGATGAAGAGCGCCATGTTGGCGAC
CTGGGCAACGTGACTGCTGACAAAGATGGTGTGGCCGATGTGTCTATTGAAGATTCTGTGATCTCA
CTGTCAGGCGACCATAGCATCATTGGCCGCACACTGGTGGTCCATGAAAAAGCAGATGACCTGGGC
AAAGGTGGCAATGAAGAAAGTACAAAAACAGGCAACGCTGGCAGTCGTCTGGCTTGTGGTGTGATT
GGTATCGCCCAAGGCGGTGGCGGTAGCCTGTGCACCCCGAGCCGTTAA
```

SUPPLEMENTARY METHODS

Simulations

We initialized the starting model for simulations by isolating the monomer in the crystal structure of the human SOD1 protein (PDB 3ECU).[1] We modified this model to match the version of SOD1 measured experimentally by removing the metal ions and disulfide bridge. We then equilibrated the initial model to alleviate crystallization and multimerization artifacts. Equilibration was performed using an all-atom force-field with explicit solvent, with the SOD1 monomer surrounded by a water box of 13,248 TIP3P water molecules and neutralized by Na^+ and Cl^- ions. Explicit-solvent, all-atom simulations with dynamics modeled by the ff14SB force-field[2] were performed using AMBER16[3], first minimizing the energy of the entire system, then heating the system to 310 K, and finally simulated the system as an isothermal-isobaric ensemble for 12 ns. The final configuration of the SOD1 monomer protein was extracted from the water box and used as the starting model for coarse-grained modeling of the pulling experiments.

Coarse-grained simulations closely followed the approach of Habibi *et al.*, who previously simulated mechanical unfolding of the loop-truncation mutant of the SOD1 monomer.[4] We used the SMOG tool[5] to perform coarse-graining, converting the all-atom structural model to a simple Cα Go-type model in which each amino acid is represented by a single bead centered at the Cα position. The dynamics of this model were computed using a force-field in which contacts (pairs of heavy atoms that are within 6 Å) have an interaction energy defined by an attractive 10-12 Lennard-Jones potential, whereas non-contact residue pairs have a repulsive interaction energy defined by a similar Lennard-Jones potential. To account for the flexibility of loops IV and VII in the absence of metal ion binding and the disulfide bond, we modified the coarse-grained force field to remove contacts from these loops, reflecting the relative lack of order.[6–8]

Pulling simulations of the coarse-grained model were done with GROMACS 5.0.7,[9] using similar parameters to Habibi *et al.*: a pulling speed of 1 m s^{-1}, a coarse-grained temperature of 140 K, a spring constant of 6 pN nm^{-1}, and a time step of 5 fs. The pulling force was applied to the N-terminus (though similar results were obtained for pulling from the C-terminus), and snapshots were saved from the simulation every 5 ps. Intermediate structures and the mechanical unfolding pathways were annotated from the simulations by manual inspection of individual structures that persist longer than 10 ps in a single trajectory. These individual structures were then classified according to the scheme in Table 1. We first tested our implementation of the method by replicating the results of Habibi *et al.* on the truncated-loop mutant SOD1 monomer, obtaining similar results for the order of events in the mechanical unfolding pathway. We then performed 26 pulling simulations on full-length SOD1 monomers; a representative unfolding simulation is shown in Figure 5.

Identifying potential intermediate structures from folding pathway analysis

To identify structural possibilities for the intermediate states, we constructed ‘substructures’ of the native SOD1 fold that consisted of some subset of the strands in the native fold retaining their structure. Each substructure thus contained at least one region of folded residues, and typically from 1 to 3 regions of unfolded residues: at the C terminus of the polypeptide chain (if C-terminal structures have unfolded), at the N terminus (if N-terminal structures have unfolded), and possibly in the middle (if there are two distinct regions of the chain that remain folded). We then compared the ΔL_c values that would be expected from transitions between these substructures to the values observed experimentally.

The substructures we included in the analysis were constrained based on several physical considerations, as described in the main text. These constraints led to 63 possibilities for consideration in the analysis. We further constrained the substructures that were included in the final analysis based on two empirical observations. First, strand 8 is known from experiment and simulation to have low stability,[10,11] hence we excluded the hairpin β7–8 as being mechanically unstable. Second, certain combinations of folded/unfolded strands do not have sufficient hydrogen bonds remaining to preserve the structure of all of the strands putatively folded, and hence were excluded. (For example, β5-7 should have three folded strands, but these strands are stabilized in the native structure by bonds with other strands that are no longer folded, and they themselves do not have a sufficiently robust hydrogen-bonding network to engender mechanical stability.) Finally, the measurements of the hairpin β2–3 peptide indicated a mechanical stability higher than that of most of the intermediate states, hence substructures that involved dissociating strands 2 and 3 early in the folding were also eliminated from the analysis.

The final list of substructures considered is shown in Supplementary Table 1. For each substructure, we calculated the expected cumulative change in L_c from the native state by adding together the total contour length of unfolded polypeptide and the end-to-end distance of each structured region in the intermediate (*i.e.*, the distance between the termini in each structured region), and then subtracting the distance between the termini in the native structure. Because the values of the contour lengths and end-to-end distances in the substructures depend on the boundaries defining the strands in the native fold, we included uncertainty arising from the native structure in the analysis. Using the structural models of SOD1 from crystallography[1] (PDB 3ECU) and solution NMR[6] (PDB 1RK7), we recomputed the strands positions for each of the 8 strands using the secondary-structure assignment program STRIDE,[12] leading to the range of cumulative ΔL_c values for each substructure listed in Supplementary Table 1.

We then examined the sequence of intermediates in each FEC, comparing the observed cumulative ΔL_c values to the range of values expected for each substructure in our list to detect all substructures that could match each intermediate state (Supplementary Figure 1). Substructures were considered to match the intermediate if the difference between predicted and observed ΔL_c values was less than a specified threshold. We used a threshold of 4 nm, to allow for error in assigning states in the FECs (the standard deviation of the peaks defining the intermediates was 2 nm, hence this criterion is equivalent to 2σ). The matching substructures were then used as nodes in a network analysis to find all possible sequential paths through the substructures that were consistent with the data, rejecting all combinations of states that could not form sequentially. In some cases, more than one path was possible, in which case each pathway was weighted by the reciprocal of the number of pathways consistent with that FEC. A weighted sum of all the pathways found for all FECs determined the most common structural transitions in the folding, allowing us to identify the most likely structures for the intermediates.

SUPPLEMENTARY REFERENCES

1. Banci, L. *et al.* Structural and dynamic aspects related to oligomerization of apo SOD1 and its mutants. *Proc. Natl. Acad. Sci. U. S. A.* **106,** 6980–6985 (2009).
2. Maier, J. A. *et al.* ff14SB: Improving the Accuracy of Protein Side Chain and Backbone Parameters from ff99SB. *J. Chem. Theory Comput.* **11,** 3696–3713 (2015).
3. Case, D. A. *et al.* The Amber biomolecular simulation programs. *J. Comput. Chem.* **26,** 1668–1688 (2005).
4. Habibi, M., Rottler, J. & Plotkin, S. S. As Simple As Possible, but Not Simpler: Exploring the Fidelity of Coarse-Grained Protein Models for Simulated Force Spectroscopy. *PLOS Comput. Biol.* **12,** e1005211 (2016).
5. Noel, J. K. *et al.* SMOG 2: A Versatile Software Package for Generating Structure-Based Models. *PLoS Comput. Biol.* **12,** (2016).
6. Banci, L., Bertini, I., Cramaro, F., Del Conte, R. & Viezzoli, M. S. Solution structure of Apo Cu,Zn superoxide dismutase: role of metal ions in protein folding. *Biochemistry* **42,** 9543–9553 (2003).
7. Furukawa, Y. *et al.* Conformational Disorder of the Most Immature Cu, Zn-Superoxide Dismutase Leading to Amyotrophic Lateral Sclerosis. *J. Biol. Chem.* **291,** 4144–4155 (2016).
8. Shaw, B. F. & Valentine, J. S. How do ALS-associated mutations in superoxide dismutase 1 promote aggregation of the protein? *Trends Biochem. Sci.* **32,** 78–85 (2007).
9. Abraham, M. J. *et al.* GROMACS: High performance molecular simulations through multi-level parallelism from laptops to supercomputers. *SoftwareX* **1,** 19–25 (2015).
10. Ding, F. & Dokholyan, N. V. Dynamical roles of metal ions and the disulfide bond in Cu, Zn superoxide dismutase folding and aggregation. *Proc. Natl. Acad. Sci. U. S. A.* **105,** 19696–19701 (2008).
11. Nordlund, A. & Oliveberg, M. Folding of Cu/Zn superoxide dismutase suggests structural hotspots for gain of neurotoxic function in ALS: parallels to precursors in amyloid disease. *Proc. Natl. Acad. Sci. U. S. A.* **103,** 10218–10223 (2006).
12. Frishman, D. & Argos, P. Knowledge-based protein secondary structure assignment. *Proteins* **23,** 566–579 (1995).
13. Sieber, V. & Moe, G. R. Interactions contributing to the formation of a beta-hairpin-like structure in a small peptide. *Biochemistry* **35,** 181–188 (1996).
14. Blanco, F. J., Rivas, G. & Serrano, L. A short linear peptide that folds into a native stable beta-hairpin in aqueous solution. *Nat. Struct. Biol.* **1,** 584–590 (1994).
15. Micsonai, A. *et al.* Accurate secondary structure prediction and fold recognition for circular dichroism spectroscopy. *Proc. Natl. Acad. Sci. U. S. A.* **112,** E3095-3103 (2015).
